# Supplementary material for: Genetic and clinical variables act synergistically to impact neurodevelopmental outcomes in children with single ventricle heart disease
Source: Commun Med (Lond). 2023 Sep 27;3:127. doi: 10.1038/s43856-023-00361-2 (PMC10533527; doi:10.1038/s43856-023-00361-2)
Supplement: Supplementary file 2 — Supplementary Information [file 43856_2023_361_MOESM2_ESM.pdf]

Supplementary Table 1. Enrollment demographics of each analytic cohort

		SVR and ISV Trial Enrollment Cohorts (n= 785)	Analytic Cohort with MDI/PDI/Length (n=304)	Cohort with full covariates (n=179)	Trios (n=81)
Female Sex (%)		36	35	38	35
Race (%)	White	80	84	88	89
	Black	15	12	9	5
	Asian	2	1	1	2
	Other	3	4	2	4
Ethncity (%)	Hispanic	17	15	14	12
	Non - hispanic	81	84	86	88
	Other	2	1	0	0
HLHS (%)	Yes	81	76	88	85
	No	19	24	12	15
Mean Gestational Age (weeks)		38.0	38.3	38.3	38.2
Mean Birthweight (grams)		3149	3187	3097	3076

Supplementary Data 1. Damaging variants in Abnormal Heart Morphology HPO-derived genes.

ID	GENE	MIM	DISEASE	INHERITANCE	LOCATION	TYPE	CSQ	CLINVAR	GEM Score	SEX	ANCESTRY	AF	GNOMAD
92020048	ABCC6	177850	PSEUDOXANTHOMA ELASTICUM, FORME FRUSTE	ad	16:16248831:G:A	SNV	missense_variant	pathogenic_het	2.145	Male	Other	0	gnomad_AF=0.00086141
92022650	ABCC6	614473	ARTERIAL CALCIFICATION, GENERALIZED, OF INFANCY, 2	ar	16:16269775:G:A	SNV	missense_variant	pathogenic_het	0.956	Male	African	0.0026419	gnomad_AF=0.00076492
92022650	ABCC6	614473	ARTERIAL CALCIFICATION, GENERALIZED, OF INFANCY, 2	ar	16:16272764:C:T	SNV	missense_variant	pathogenic_het	0.956	Male	African	0.0025241	gnomad_AF=0.00070073
92026925	ABCC6	177850	PSEUDOXANTHOMA ELASTICUM, FORME FRUSTE	ad	16:16256935:G:A	SNV	stop_gained	pathogenic_het	1.907	Female	European(non-Finish)	0.0020086	gnomad_AF=0.0013053
GT04007462	ABCC6	177850	PSEUDOXANTHOMA ELASTICUM, FORME FRUSTE	ad	16:16256935:G:A	SNV	stop_gained	pathogenic_het	2.048	Male	European(non-Finish)	0.0020086	gnomad_AF=0.0013053
GT04012012	ABCC6	177850	PSEUDOXANTHOMA ELASTICUM, FORME FRUSTE	ad	16:16255422:C:T	SNV	splice_acceptor_variant	likely_pathogenic_het	2.316	Male	Other	0.0011792	gnomad_AF=3.1851e-05
9504000218-0	ACTA2	611788	AORTIC ANEURYSM, FAMILIAL THORACIC 6	ad	10:90701089:A:T	SNV	stop_gained	VUS	1.491	Female	Other	0	no AF in gnomad
GT04013912	ACTG2	155310	VISCERAL MYOPATHY	ad	2:74141940:C:G	SNV	missense_variant	VUS	1.044	Male	European(non-Finish)	0	no AF in gnomad
92123604	ADAR	127400	DYSCHROMATOSIS SYMMETRICA HEREDITARIA	ad	1:154557514:C:A	SNV	missense_variant	VUS	0.754	Female	Finish	0	no AF in gnomad
92021355	AFF4	616368	CHOPS SYNDROME	ad	5:132220818:A:G	SNV	splice_region_variant,intron_variant	VUS	0.79	Male	European(non-Finish)	0	no AF in gnomad
92020074	ANKRD11	148050	KBG SYNDROME	ad	16:89347773:G:A	SNV	missense_variant	VUS	0.986	Male	European(non-Finish)	0	no AF in gnomad
92026418	ANKRD11	148050	KBG SYNDROME	ad	16:89349242:CTGTT	INDEL	frameshift_variant	pathogenic_het	3.124	Female	European(non-Finish)	0	NA
GT04007303	ANKRD11	148050	KBG SYNDROME	ad	16:89350282:G:A	SNV	missense_variant	VUS	0.731	Male	European(non-Finish)	0	no AF in gnomad
9504000235-0	ANO5	166260	GNATHODIAPHYSEAL DYSPLASIA	ad	11:22291960:TG:T	INDEL	frameshift_variant	pathogenic_het	2.342	Female	European(non-Finish)	0	no AF in gnomad
GT04013282	APOB	144010	HYPERCHOLESTEROLEMIA, FAMILIAL, 2	ad	2:21232182:G:A	SNV	stop_gained	VUS	0.891	Female	Other	0	no AF in gnomad
92023145	ARHGAP31	100300	ADAMS-OLIVER SYNDROME 1	ad	3:119087363:G:A	SNV	splice_region_variant,synonymous_variant	VUS	0.861	Male	Other	0	gnomad_AF=3.1865e-05
92121092	ARID1A	614607	COFFIN-SIRIS SYNDROME 2	ad	1:27024018:C:A	SNV	missense_variant	VUS	1.056	Male	European(non-Finish)	0	no AF in gnomad
92024159	ARVCF	NA	NA	ad	22:19965033:T:C	SNV	missense_variant	VUS	0.807	Male	African	0	no AF in gnomad
92027460	ATN1	618494	CONGENITAL HYPOTONIA, EPILEPSY, DEVELOPMENTAL DELAY, AND DIGITAL ANOMALIES	ad	12:7047234:A:C	SNV	splice_region_variant,intron_variant	VUS	0.734	Male	Finish	0	no AF in gnomad
9504000131-0	BCOR	300166	MCOPS2 SYNDROME	xd	X:39937132:G:C	SNV	missense_variant	VUS	0.795	Female	European(non-Finish)	0	gnomad_AF=4.5212e-05
GT04007522	BCOR	300166	MCOPS2 SYNDROME	xd	X:39937132:G:C	SNV	missense_variant	VUS	0.776	Female	European(non-Finish)	0	gnomad_AF=4.5212e-05
GT04011762	BCOR	300166	MCOPS2 SYNDROME	xd	X:39937132:G:C	SNV	missense_variant	VUS	0.784	Female	European(non-Finish)	0	gnomad_AF=4.5212e-05
92120381	BICC1	601331	RENAL DYSPLASIA, CYSTIC, SUSCEPTIBILITY TO	ad	10:60566890:A:G	SNV	missense_variant	VUS	0.711	Male	Other	0	no AF in gnomad
GT04011222	BICC1	601331	RENAL DYSPLASIA, CYSTIC, SUSCEPTIBILITY TO	ad	10:60566890:A:G	SNV	missense_variant	VUS	0.752	Male	Other	0	no AF in gnomad
GT04011733	BTK	300755	AGAMMAGLOBULINEMIA, X-LINKED	xr	X:100612542:A:G	SNV	missense_variant	VUS	0.795	Male	European(non-Finish)	0	no AF in gnomad
92021090	CDH23	617540	PITUITARY ADENOMA 5, MULTIPLE TYPES	ad	10:73565608:A:T	SNV	stop_gained	VUS	0.918	Male	European(non-Finish)	0	no AF in gnomad
GT04011562	CDK13	617360	CONGENITAL HEART DEFECTS, DYSMORPHIC FACIAL FEATURES, AND INTELLECTUAL DEVELOPMENTAL DISORDER	ad	7:40132548:G:T	SNV	stop_gained	VUS	1.129	Female	Other	0	no AF in gnomad
GT04014731	CDK8	618748	INTELLECTUAL DEVELOPMENTAL DISORDER WITH HYPOTONIA AND BEHAVIORAL ABNORMALITIES	ad	13:26927875:A:C	SNV	splice_acceptor_variant	VUS	1.393	Male	European(non-Finish)	0	no AF in gnomad
9504000056-0	CEP290	611134	MECKEL SYNDROME, TYPE 4	ad	12:88477631:G:A	SNV	missense_variant	likely_pathogenic_het	1.255	Male	Other	0	gnomad_AF=6.3739e-05
9504000303-0	CHD7	214800	CHARGE SYNDROME	ad	8:61777576:G:T	SNV	missense_variant,splice_region_variant	VUS	0.839	Male	Other	0	no AF in gnomad
92120189	CHN1	604356	DUANE RETRACTION SYNDROME 2	ad	2:175673698:T:G	SNV	missense_variant	VUS	1.423	Male	European(non-Finish)	0	no AF in gnomad
92024262	CLIP2	NA	NA	ad	7:73790841:C:T	SNV	missense_variant	VUS	0.81	Male	Ashkenazi	0	no AF in gnomad
92026608	CLIP2	NA	NA	ad	7:73774562:C:G	SNV	missense_variant	VUS	0.969	Male	European(non-Finish)	0	no AF in gnomad
GT04006123	CLIP2	NA	NA	ad	7:73731907:G:A	SNV	missense_variant	VUS	0.754	Female	Ashkenazi	0	no AF in gnomad
GT04012081	COG4	618150	SAUL-WILSON SYNDROME	ad	16:70516712:C:A	SNV	stop_gained	pathogenic_het	2.744	Female	Asian	0	gnomad_AF=6.3914e-05
GT04010132	COL11A1	228520	FIBROCHONDROGENESIS 1	ad	1:103345432:A:T	SNV	missense_variant	VUS	0.832	Male	European(non-Finish)	0	no AF in gnomad
GT04006123	CR2	240500	IMMUNODEFICIENCY, COMMON VARIABLE, 2	ad	1:207646887:C:T	SNV	splice_region_variant,intron_variant	VUS	0.891	Female	Ashkenazi	0	no AF in gnomad
92029111	CREBBP	180849	BROAD THUMBS AND GREAT TOES, CHARACTERISTIC FACIES, AND MENTAL RETARDATION	ad	16:3778101:G:C	SNV	missense_variant	VUS	0.802	Male	Other	0	no AF in gnomad
GT04011562	CRKL	NA	NA	ad	22:21288342:A:G	SNV	missense_variant	VUS	0.882	Female	Other	0	gnomad_AF=3.1849e-05
92025048	CRYAB	608810	MYOPATHY, MYOFIBRILLAR, 2	ad	11:111781100:T:C	SNV	missense_variant	VUS	0.706	Male	African	0	no AF in gnomad
92120557	CRYAB	608810	MYOPATHY, MYOFIBRILLAR, 2	ad	11:111781100:T:C	SNV	missense_variant	VUS	0.744	Male	African	0	no AF in gnomad
92024159	CTLA4	152700	SYSTEMIC LUPUS ERYTHEMATOSUS	ad	2:204735440:G:A	SNV	missense_variant	VUS	0.883	Male	African	0	no AF in gnomad
92027345	DACT1	617466	TOWNES-BROCKS SYNDROME 2	ad	14:59113613:G:A	SNV	missense_variant	VUS	0.787	Male	European(non-Finish)	0	no AF in gnomad
9504000101-0	DCC	157600	AGENESIS OF CORPUS CALLOSUM	ad	18:50985658:C:T	SNV	missense_variant	VUS	0.732	Female	African	0	no AF in gnomad
GT04012081	DCC	157600	AGENESIS OF CORPUS CALLOSUM	ad	18:50866178:G:A	SNV	missense_variant	pathogenic_het	1.707	Female	Asian	0.0025641	gnomad_AF=0.00019116
92021937	DCHS1	607829	MITRAL VALVE PROLAPSE 2	ad	11:6654183:G:A	SNV	missense_variant	VUS	0.723	Female	African	0	no AF in gnomad
92021937	DSP	605676	CARDIOMYOPATHY, DILATED, WITH WOOLLY HAIR AND KERATODERMA	ad	6:7565658:G:A	SNV	missense_variant	VUS	0.814	Female	African	0	no AF in gnomad
92123565	DSP	605676	CARDIOMYOPATHY, DILATED, WITH WOOLLY HAIR AND KERATODERMA	ad	6:7584815:GCT:G	INDEL	frameshift_variant	VUS	1.202	Male	European(non-Finish)	0	no AF in gnomad
9504000065-0	DSP	605676	CARDIOMYOPATHY, DILATED, WITH WOOLLY HAIR AND KERATODERMA	ad	6:7569464:G:A	SNV	missense_variant	VUS	0.791	Male	European(non-Finish)	0	no AF in gnomad
9504000325-0	DTNA	604169	LEFT VENTRICULAR NONCOMPACTION 1	ad	18:32462094:G:T	SNV	missense_variant	likely_pathogenic_het	2.65	Female	Other	0	no AF in gnomad
9504000309-0	DVL3	616894	ROBINOW SYNDROME, AUTOSOMAL DOMINANT 3	ad	3:183883971:G:T	SNV	missense_variant	VUS	0.728	Female	European(non-Finish)	0	no AF in gnomad
9504000309-0	DVL3	616894	ROBINOW SYNDROME, AUTOSOMAL DOMINANT 3	ad	3:183883971:G:T	SNV	missense_variant	VUS	0.731	Female	European(non-Finish)	0	no AF in gnomad
9504000182-0	ECE1	613870	HIRSCHSPRUNG DISEASE, CARDIAC DEFECTS, AND AUTONOMIC DYSFUNCTION	ad	1:21551931:G:A	SNV	missense_variant,splice_region_variant	VUS	0.738	Male	European(non-Finish)	0	no AF in gnomad
92021393	EED	617561	COHEN-GIBSON SYNDROME	ad	11:85956306:G:C	SNV	missense_variant	VUS	1.448	Male	European(non-Finish)	0	no AF in gnomad
92022282	EFTUD2	610536	MANDIBULOFACIAL DYSOSTOSIS, GUION-ALMEIDA TYPE	ad	17:42931700:C:T	SNV	missense_variant	VUS	1.303	Female	European(non-Finish)	0	no AF in gnomad
9504000092-0	ELN	194050	WILLIAMS-BEUREN SYNDROME	ad	7:73477952:C:T	SNV	splice_region_variant,synonymous_variant	VUS	1.022	Male	European(non-Finish)	0	no AF in gnomad
92021141	EP300	613684	RUBENSTEIN-TAYBI TYPE 2	ad	22:41574281:TGCAA	INDEL	inframe_deletion	likely_pathogenic_het	1.818	Male	Ashkenazi	0	NA
GT04010662	EXT1	133700	EXOSTOSES, MULTIPLE, TYPE I	ad	8:119122874:T:A	SNV	missense_variant	VUS	0.968	Male	European(non-Finish)	0	no AF in gnomad
92025579	EZH2	277590	WEAVER-LIKE SYNDROME	ad	7:148507504:A:G	SNV	splice_region_variant,synonymous_variant	VUS	0.948	Male	African	0	no AF in gnomad
GT04011822	FANCB	314390	VACTERL ASSOCIATION, X-LINKED, WITH OR WITHOUT HYDROCEPHALUS	xr	X:14883175:A:T	SNV	missense_variant	VUS	0.808	Male	Other	0	no AF in gnomad
92120824	FANCG	NA	NA	ad	9:35076529:C:A	SNV	stop_gained	VUS	1.174	Female	Other	0	gnomad_AF=3.1863e-05
92020062	FBLN5	614434	CUTIS LAXA, AUTOSOMAL DOMINANT	ad	14:92343929:C:T	SNV	missense_variant	pathogenic_het	1.182	Male	European(non-Finish)	6.48E-05	gnomad_AF=3.1851e-05
9504000115-0	FBN1	154700	MARFAN SYNDROME	ad	15:48766740:C:T	SNV	missense_variant	VUS	0.98	Female	European(non-Finish)	0	no AF in gnomad
9504000229-0	FBN2	121050	CONTRACTURAL ARACHNODACTYLY, CONGENITAL	ad	5:127641211:G:T	SNV	missense_variant	VUS	0.872	Female	European(non-Finish)	0	no AF in gnomad
92022422	FGD1	305400	AARSKOG-SCOTT SYNDROME	xr	X:54472639:G:A	SNV	missense_variant	VUS	1.041	Male	European(non-Finish)	0	no AF in gnomad
GT04012142	FHL1	300280	URUGUAY FACIOCARDIOMUSCULOSKELETAL SYNDROME	xr	X:135291407:AG:A	INDEL	frameshift_variant	VUS	1.487	Male	European(non-Finish)	0	no AF in gnomad
9504000218-0	FLRT3	146110	HYPOGONADOTROPIC HYPOGONADISM 7 WITH OR WITHOUT ANOSMIA	ad	20:14306708:A:T	SNV	missense_variant	VUS	1.195	Female	Other	0	no AF in gnomad
92021052	FOS	NA	NA	ad	14:75746823:G:A	SNV	missense_variant	VUS	1.089	Male	European(non-Finish)	0	no AF in gnomad
9504000274-0	GJA1	164200	OCULODENTODIGITAL DYSPLASIA	ad	6:121769125:G:A	SNV	missense_variant	VUS	0.921	Male	Ashkenazi	0	no AF in gnomad
9504000008-0	GLI3	146510	PALLISTER-HALL SYNDROME	ad	7:42005606:G:A	SNV	missense_variant	VUS	0.916	Male	European(non-Finish)	0	no AF in gnomad
9504000367-0	GNE	269921	SIALURIA	ad	9:36218221:G:A	SNV	missense_variant	pathogenic_het	1.412	Male	European(non-Finish)	6.49E-05	gnomad_AF=6.3743e-05
92028878	GSN	105120	AMYLOIDOSIS V	ad	9:124086871:T:TGC	INDEL	frameshift_variant	VUS	0.755	Female	Other	0	NA
9504000244-0	GTF2I	194050	WILLIAMS-BEUREN SYNDROME	ad	7:74131206:C:A	SNV	splice_region_variant,intron_variant	VUS	0.699	Male	European(non-Finish)	0	no AF in gnomad
92023258	GTF2IRD1	NA	NA	ad	7:73932531:G:A	SNV	missense_variant	VUS	1.077	Female	European(non-Finish)	0	no AF in gnomad
92029301	GTF2IRD1	NA	NA	ad	7:73922429:C:T	SNV	missense_variant	VUS	0.718	Male	Asian	0	gnomad_AF=3.1878e-05
GT04011882	GTF2IRD1	NA	NA	ad	7:73972004:G:A	SNV	missense_variant	VUS	0.85	Male	European(non-Finish)	0	no AF in gnomad
9504000014-0	HCN4	163800	SICK SINUS SYNDROME 2	ad	15:73617719:C:T	SNV	missense_variant	pathogenic_het	2.392	Male	Ashkenazi	0	no AF in gnomad
9504000051-0	HIC1	NA	NA	ad	17:1959641:G:A	SNV	splice_donor_variant	VUS	0.766	Female	African	0.0003445	gnomad_AF=9.5669e-05
92020048	HNRNPA1	615426	AMYOTROPHIC LATERAL SCLEROSIS 20	ad	12:54676358:T:C	SNV	splice_region_variant,synonymous_variant	VUS	0.692	Male	Other	0	no AF in gnomad
92024147	HOXD13	186000	SYNPOLYDACTYLY 1	ad	2:176957822:G:GCG	INDEL	frameshift_variant	VUS	0.801	Male	European(non-Finish)	0	NA
9504000303-0	IFIH1	182250	SINGLETON-MERTEN SYNDROME 1	ad	2:163128888:G:A	SNV	stop_gained	VUS	0.697	Male	Other	0	gnomad_AF=6.3723e-05
GT04012532	IKBKG	300636	IMMUNODEFICIENCY 33	xr	X:153780339:T:G	SNV	missense_variant	VUS	1.022	Male	Ashkenazi	0	no AF in gnomad
GT04013282	IL17RD	146110	HYPOGONADOTROPIC HYPOGONADISM 7 WITH OR WITHOUT ANOSMIA	ad	3:57139956:C:T	SNV	missense_variant	likely_pathogenic_het	2.434	Female	Other	0	no AF in gnomad
92021216	INPP5E	213300	CPD4	ar	9:139327608:T:TCC	INDEL	inframe_insertion,splice_region_variant	VUS	0.775	Male	African	0	NA
92021216	INPP5E	213300	CPD4	ar	9:139327605:A:ACC	INDEL	splice_donor_variant	VUS	0.775	Male	African	0	gnomad_AF=3.2571e-05
92022282	INS	606176	DIABETES MELLITUS, PERMANENT NEONATAL, WITH NEUROLOGIC FEATURES	ad	11:2181203:C:A	SNV	missense_variant	VUS	0.907	Female	European(non-Finish)	0	no AF in gnomad
9504000194-0	IQSEC2	309530	INTELLECTUAL DEVELOPMENTAL DISORDER, X-LINKED 1	xd	X:53263715:G:A	SNV	missense_variant	VUS	1.356	Female	Other	0	no AF in gnomad
92022371	ITGA8	191830	RENAL HYPODYSPLASIA/APLASIA 1	ad	10:15617485:T:C	SNV	splice_region_variant,intron_variant	VUS	0.987	Male	African	0	no AF in gnomad
92021735	KCNH1	135500	LABAND SYNDROME	ad	1:210857029:T:C	SNV	missense_variant	VUS	1.131	Male	European(non-Finish)	0	no AF in gnomad
9504000024-0	KISS1R	176400	PRECOCIOUS PUBERTY, CENTRAL, 1	ad	19:919929:GCGCGCC	INDEL	inframe_deletion	VUS	1.219	Male	Ashkenazi	0	NA
92027876	KMT2A	605130	WIEDEMANN-STEINER SYNDROME	ad	11:118374319:G:A	SNV	missense_variant	VUS	0.844	Male	European(non-Finish)	0	no AF in gnomad
9504000368-0	KMT2A	605130	WIEDEMANN-STEINER SYNDROME	ad	11:118374568:C:T	SNV	missense_variant	VUS	0.833	Male	European(non-Finish)	0	no AF in gnomad
GT04011592	KMT2A	605130	WIEDEMANN-STEINER SYNDROME	ad	11:118374319:G:A	SNV	missense_variant	VUS	0.892	Male	European(non-Finish)	0	no AF in gnomad
92120189	KMT2D	147920	KABUKI SYNDROME 1	ad	12:49445067:TGA:T	INDEL	frameshift_variant	VUS	1.086	Male	European(non-Finish)	0	no AF in gnomad
9504000274-0	KMT2D	147920	KABUKI SYNDROME 1	ad	12:49420510:ACATT	INDEL	frameshift_variant	likely_pathogenic_het	3.363	Male	Ashkenazi	0	NA
GT04008173	KMT2D	147920	KABUKI SYNDROME 1	ad	12:49446763:A:T	SNV	stop_gained	VUS	1.22	Female	European(non-Finish)	0	no AF in gnomad
GT04010993	KMT2D	147920	KABUKI SYNDROME 1	ad	12:49447337:G:GC	INDEL	frameshift_variant	VUS	1.019	Male	European(non-Finish)	0	no AF in gnomad
GT04012081	KMT2D	147920	KABUKI SYNDROME 1	ad	12:49420288:C:T	SNV	missense_variant	pathogenic_het	2.701	Female	Asian	0	no AF in gnomad
9504000147-0	LAMB3	104530	AMELOGENESIS IMPERFECTA, TYPE IA	ad	1:209799066:G:A	SNV	stop_gained	pathogenic_het	1.941	Male	European(non-Finish)	0.0007127	gnomad_AF=0.00041401
92025175	LDB3	601493	CARDIOMYOPATHY, DILATED, 1C, WITH OR WITHOUT LEFT VENTRICULAR NONCOMPACTION	ad	10:88441257:GCCCA	INDEL	inframe_deletion	VUS	0.698	Male	European(non-Finish)	0	NA
9504000194-0	LETM1	194190	OMIM:194190	ad	4:1824753:G:A	SNV	missense_variant	VUS	1.201	Female	Other	0	no AF in gnomad
92120937	LRP5	259770	OSTEOPOROSIS-PSEUDOGLIOMA SYNDROME	ad	11:68131215:G:A	SNV	splice_region_variant,synonymous_variant	VUS	0.823	Male	European(non-Finish)	0	no AF in gnomad
92023602	LZTR1	616564	NOONAN SYNDROME 10	ad	22:21343948:C:T	SNV	stop_gained	pathogenic_het	2.209	Female	European(non-Finish)	0.0001298	gnomad_AF=9.5669e-05
92026608	MAP1B	618918	PERIVENTRICULAR NODULAR HETEROTOPIA 9	ad	5:71496062:G:A	SNV	missense_variant	VUS	1.086	Male	European(non-Finish)	0	no AF in gnomad
9504000269-0	MAP3K1	613762	46,XY SEX REVERSAL 6	ad	5:56178149:A:T	SNV	missense_variant	VUS	0.722	Male	European(non-Finish)	0	no AF in gnomad
GT04007861	MED12	305450	OPITZ-KAVEGGIA SYNDROME	xr	X:70357766:A:G	SNV	missense_variant	VUS	1.219	Male	European(non-Finish)	0	no AF in gnomad
GT04012732	MED12	305450	OPITZ-KAVEGGIA SYNDROME	xr	X:70344633:C:G	SNV	missense_variant	VUS	0.768	Male	European(non-Finish)	0	no AF in gnomad
9504000194-0	MEIS2	600987	CLEFT PALATE, CARDIAC DEFECTS, AND MENTAL RETARDATION	ad	15:37184453:A:G	SNV	missense_variant	VUS	1.332	Female	Other	0	no AF in gnomad
9504000322-0	MIB1	615092	LEFT VENTRICULAR NONCOMPACTION 7	ad	18:19353605:G:C	SNV	missense_variant	VUS	0.792	Male	European(non-Finish)	0	no AF in gnomad
92120418	MMP21	616749	HETEROTAXY, VISCERAL, 7, AUTOSOMAL	ar	10:127464229:C:A	SNV	missense_variant,splice_region_variant	VUS	0.731	Female	Other	0	no AF in gnomad
92120418	MMP21	616749	HETEROTAXY, VISCERAL, 7, AUTOSOMAL	ar	10:127464224:C:G	SNV	splice_region_variant,intron_variant	VUS	0.731	Female	Other	0	no AF in gnomad
92120367	MPL	254450	MYELOFIBROSIS	ad	1:43804304:C:T	SNV	missense_variant	pathogenic_het	2.962	Male	European(non-Finish)	0	no AF in gnomad
92124125	MSH6	614350	COLORECTAL CANCER, HEREDITARY NONPOLYPOSIS, TYPE 5	ad	2:48030639:A:AC	INDEL	frameshift_variant	pathogenic_het	2.499	Male	European(non-Finish)	0	gnomad_AF=3.2128e-05
9504000200-0	MYBPC3	115197	CARDIOMYOPATHY, FAMILIAL HYPERTROPHIC, 4	ad	11:47364129:C:G	SNV	missense_variant,splice_region_variant	pathogenic_het	1.776	Male	European(non-Finish)	0.0001297	gnomad_AF=6.3747e-05
9504000383-0	MYH11	132900	AORTIC ANEURYSM, FAMILIAL THORACIC 4	ad	16:15810993:A:C	SNV	splice_region_variant,intron_variant	VUS	0.838	Female	European(non-Finish)	0	no AF in gnomad
9504000068-0	MYLK	613780	AORTIC ANEURYSM, FAMILIAL THORACIC 7	ad	3:123454265:T:C	SNV	splice_acceptor_variant	VUS	0.721	Male	European(non-Finish)	0	no AF in gnomad
92020062	MYRF	618280	CARDIAC-UROGENITAL SYNDROME	ad	11:61539309:C:T	SNV	stop_gained	VUS	1.495	Male	European(non-Finish)	0	no AF in gnomad
GT04010762	NADSYN1	618845	VERTEBRAL, CARDIAC, RENAL, AND LIMB DEFECTS SYNDROME 3	ar	11:71202902:G:A	SNV	missense_variant	pathogenic_hom	1.543	Male	European(non-Finish)	0.0023995	gnomad_AF=0.0012108
9504000108-0	NEUROD2	618374	EPILEPTIC ENCEPHALOPATHY, EARLY INFANTILE, 72	ad	17:37762310:C:G	SNV	missense_variant	VUS	1.004	Female	Other	0	no AF in gnomad
9504000200-0	NF1	193520	WATSON SYNDROME	ad	17:29553448:C:T	SNV	splice_region_variant,intron_variant	VUS	0.839	Male	European(non-Finish)	0	no AF in gnomad
9504000072-0	NFKB1	616576	IMMUNODEFICIENCY, COMMON VARIABLE, 12	ad	4:103531776:G:A	SNV	missense_variant	VUS	0.754	Male	European(non-Finish)	0	no AF in gnomad
9504000229-0	NIPBL	122470	BDLS	ad	5:37022498:T:G	SNV	splice_region_variant,intron_variant	VUS	0.774	Female	European(non-Finish)	0	no AF in gnomad
92021521	NONO	300967	MENTAL RETARDATION, X-LINKED, SYNDROMIC 34	xd	X:70514199:G:C	SNV	missense_variant	VUS	0.82	Female	European(non-Finish)	0	no AF in gnomad
9504000084	NOTCH1	616028	AORTIC VALVE DISEASE 1	ad	9:139401755:AC:A	INDEL	splice_donor_variant	VUS	1.049	Male	African	0	no AF in gnomad
9504000111-0	NOTCH1	616028	AORTIC VALVE DISEASE 1	ad	9:139399785:G:T	SNV	stop_gained	VUS	1.13	Female	European(non-Finish)	0	no AF in gnomad
9504000232-0	NOTCH1	616028	AORTIC VALVE DISEASE 1	ad	9:139392011:C:A	SNV	splice_acceptor_variant	VUS	0.983	Male	European(non-Finish)	0	no AF in gnomad
9504000280-0	NOTCH1	616028	AORTIC VALVE DISEASE 1	ad	9:139417442:G:A	SNV	missense_variant	VUS	0.993	Female	European(non-Finish)	0	no AF in gnomad
9504000206-0	NR2F2	615779	CONGENITAL HEART DEFECTS, MULTIPLE TYPES, 4	ad	15:96880841:CA:C	INDEL	frameshift_variant	VUS	1.283	Female	European(non-Finish)	0	no AF in gnomad
9504000235-0	NR5A1	612965	46,XY SEX REVERSAL 3	ad	9:127265367:G:A	SNV	missense_variant	VUS	0.919	Female	European(non-Finish)	0	no AF in gnomad
9504000094-0	NSD1	117550	SOTOS SYNDROME	ad	5:176637132:G:C	SNV	missense_variant	VUS	0.82	Male	Other	0	no AF in gnomad
9504000151-0	NSMF	614838	HYPOGONADOTROPIC HYPOGONADISM 9 WITH OR WITHOUT ANOSMIA	ad	9:140348897:T:TG	INDEL	splice_region_variant,intron_variant	VUS	1.087	Male	European(non-Finish)	0	no AF in gnomad
GT04010722	OFD1	311200	OROFACIODIGITAL SYNDROME I	xd	X:13764945:CA:C	INDEL	frameshift_variant	pathogenic_het	2.12	Male	European(non-Finish)	0	gnomad_AF=0
9504000014-0	OTX2	610125	MICROPHTHALMIA, SYNDROMIC 5	ad	14:57271039:C:T	SNV	missense_variant	VUS	0.948	Male	Ashkenazi	0	no AF in gnomad
GT04008811	PBX1	617641	CONGENITAL ANOMALIES OF KIDNEY AND URINARY TRACT SYNDROME WITH OR WITHOUT HEARING LOSS, ABNORMAL EARS, OR DEVELOPMENTAL DELAY	ad	1:164790855:G:A	SNV	missense_variant	VUS	1.228	Male	Other	0	no AF in gnomad
92021381	PCGF2	618371	TURNPENNY-FRY SYNDROME	ad	17:36895914:C:T	SNV	missense_variant	VUS	0.982	Male	African	0	gnomad_AF=3.1876e-05
92020896	PCSK9	603776	HYPERCHOLESTEROLEMIA, FAMILIAL, 3	ad	1:55517946:G:C	SNV	splice_region_variant,intron_variant	VUS	0.747	Male	Other	0	no AF in gnomad
GT04008261	PIGT	615398	MULTIPLE CONGENITAL ANOMALIES-HYPOTONIA-SEIZURES SYNDROME 3	ad	20:44054311:G:A	SNV	missense_variant	likely_pathogenic_het	0.873	Male	European(non-Finish)	6.48E-05	gnomad_AF=0.00012741
9504000065-0	PKD1	173900	POLYCYSTIC KIDNEY DISEASE 1	ad	16:2147867:A:C	SNV	splice_donor_variant	VUS	0.786	Male	European(non-Finish)	0	no AF in gnomad
92027294	PKP2	609040	ARRHYTHMOGENIC RIGHT VENTRICULAR DYSPLASIA, FAMILIAL, 9	ad	12:32949233:C:T	SNV	splice_acceptor_variant	likely_pathogenic_het	2.997	Male	African	0	no AF in gnomad
92120874	PMS2	614337	COLORECTAL CANCER, HEREDITARY NONPOLYPOSIS, TYPE 4	ad	7:6035203:ACTGT:A	INDEL	frameshift_variant	pathogenic_het	2.518	Male	African	0	gnomad_AF=3.1849e-05
92022282	POGZ	616364	WHITE-SUTTON SYNDROME	ad	1:151377567:C:G	SNV	missense_variant	VUS	0.981	Female	European(non-Finish)	0	no AF in gnomad
92024147	POLR1A	616462	ACROFACIAL DYSOSTOSIS, CINCINNATI TYPE	ad	2:86255740:A:C	SNV	missense_variant	VUS	0.851	Male	European(non-Finish)	0	no AF in gnomad
GT04006052	PPP2R5D	616355	MENTAL RETARDATION, AUTOSOMAL DOMINANT 35	ad	6:42976209:C:T	SNV	missense_variant	VUS	0.744	Male	European(non-Finish)	0	no AF in gnomad
GT04015662	PROK2	610628	HYPOGONADOTROPIC HYPOGONADISM 4 WITH OR WITHOUT ANOSMIA	ad	3:71830676:AT:A	INDEL	frameshift_variant	pathogenic_het	2.253	Male	Other	0	gnomad_AF=6.371e-05
GT04008002	PRRX1	202650	AGNATHIA-OTOCEPHALY COMPLEX	ad	1:170695508:G:A	SNV	missense_variant	VUS	0.768	Male	Other	0	no AF in gnomad
9504000095-0	PTCH2	109400	BASAL CELL NEVUS SYNDROME	ad	1:45295203:C:G	SNV	splice_region_variant,synonymous_variant	VUS	0.729	Female	European(non-Finish)	0	no AF in gnomad
92020961	RET	209880	CENTRAL HYPOVENTILATION SYNDROME, CONGENITAL	ad	10:43607678:C:G	SNV	splice_region_variant,intron_variant	VUS	0.822	Male	European(non-Finish)	0	no AF in gnomad
92029301	RET	209880	CENTRAL HYPOVENTILATION SYNDROME, CONGENITAL	ad	10:43614996:G:A	SNV	missense_variant	pathogenic_het	2.055	Male	Asian	0	gnomad_AF=0.00022333
GT04013812	RET	209880	CENTRAL HYPOVENTILATION SYNDROME, CONGENITAL	ad	10:43619259:G:A	SNV	splice_region_variant,intron_variant	VUS	1.009	Female	European(non-Finish)	0	no AF in gnomad
9504000282-0	RFC2	NA	NA	ad	7:73657477:G:A	SNV	splice_region_variant,synonymous_variant	VUS	1.168	Male	Other	0	gnomad_AF=3.1845e-05
GT04014901	RREB1	NA	NA	ad	6:7248887:G:T	SNV	missense_variant	VUS	1.212	Female	European(non-Finish)	0	no AF in gnomad
GT04011402	RYR1	255310	MYOPATHY, CONGENITAL, WITH FIBER-TYPE DISPROPORTION	ad	19:39013851:C:G	SNV	splice_region_variant,intron_variant	pathogenic_het	2.302	Female	African	0.0004589	gnomad_AF=0.00012741
GT04012532	RYR1	255310	MYOPATHY, CONGENITAL, WITH FIBER-TYPE DISPROPORTION	ad	19:39019237:A:T	SNV	splice_acceptor_variant	VUS	0.777	Male	Ashkenazi	0	no AF in gnomad
GT04013872	SALL4	607323	DUANE ANOMALY WITH RADIAL RAY ABNORMALITIES AND DEAFNESS	ad	20:50401093:A:T	SNV	missense_variant	VUS	0.846	Male	African	0	no AF in gnomad
92120836	SAMHD1	614415	CHILBLAIN LUPUS 2	ad	20:35580045:A:T	SNV	start_lost	VUS	1.371	Female	European(non-Finish)	0	no AF in gnomad
92022042	SCN5A	614022	ATRIAL FIBRILLATION, FAMILIAL, 10	ad	3:38622661:C:A	SNV	missense_variant	pathogenic_het	1.641	Female	European(non-Finish)	6.50E-05	gnomad_AF=3.1922e-05
9504000149-0	SCNN1B	177200	LIDDLE SYNDROME 1	ad	16:23379281:G:A	SNV	splice_donor_variant	VUS	0.733	Male	European(non-Finish)	0	no AF in gnomad
92121129	SEC24C	NA	NA	ad	10:75528652:C:T	SNV	missense_variant	VUS	1.125	Female	European(non-Finish)	0	no AF in gnomad
92120189	SHANK3	606232	PHELAN-MCDERMID SYNDROME	ad	22:51133238:C:T	SNV	missense_variant	VUS	0.734	Male	European(non-Finish)	0	no AF in gnomad
9504000056-0	SHANK3	606232	PHELAN-MCDERMID SYNDROME	ad	22:51160651:C:T	SNV	missense_variant	VUS	0.734	Male	Other	0	no AF in gnomad
9504000182-0	SHH	142945	HOLOPROSENCEPHALY 3	ad	7:155596052:G:A	SNV	missense_variant	VUS	1.14	Male	European(non-Finish)	0	no AF in gnomad
92122373	SIM1	NA	NA	ad	6:100896458:C:A	SNV	missense_variant	VUS	1.447	Female	European(non-Finish)	0	no AF in gnomad
9504000139-0	SIM1	NA	NA	ad	6:100838928:G:A	SNV	missense_variant	VUS	1.184	Female	African	0	no AF in gnomad
GT04008362	SIM1	NA	NA	ad	6:100896458:C:A	SNV	missense_variant	VUS	1.463	Female	European(non-Finish)	0	no AF in gnomad
GT04008021	SLC25A24	612289	GORLIN-CHAUDHRY-MOSS SYNDROME	ad	1:108700128:G:A	SNV	stop_gained	VUS	1.384	Female	European(non-Finish)	0	no AF in gnomad
92020896	SLC25A4	617184	MITOCHONDRIAL DNA DEPLETION SYNDROME 12A (CARDIOMYOPATHIC TYPE), AUTOSOMAL DOMINANT	ad	4:186067983:C:T	SNV	missense_variant	VUS	0.886	Male	Other	0	no AF in gnomad
GT04010132	SLX4	613951	FANCONI ANEMIA, COMPLEMENTATION GROUP P	ar	16:3641280:C:T	SNV	missense_variant	VUS	1.019	Male	European(non-Finish)	0.0016848	gnomad_AF=0.0014013
92025048	SMAD3	613795	LOEYS-DIETZ SYNDROME 3	ad	15:67358499:T:A	SNV	missense_variant	VUS	0.891	Male	African	0	no AF in gnomad
92029147	SMC1A	300590	CORNELIA DE LANGE SYNDROME 2	xr	X:53438775:T:C	SNV	missense_variant	VUS	1.369	Male	European(non-Finish)	0	no AF in gnomad
9504000092-0	SOS1	610733	NOONAN SYNDROME 4	ad	2:39234290:A:T	SNV	missense_variant	VUS	0.898	Male	European(non-Finish)	0	no AF in gnomad
92020947	SPTA1	130600	ELLIPTOCYTOSIS 2	ad	1:158589121:G:A	SNV	missense_variant	likely_pathogenic_het	1.124	Male	European(non-Finish)	0.0030476	gnomad_AF=0.0027721
GT04006072	SPTA1	130600	ELLIPTOCYTOSIS 2	ad	1:158589121:G:A	SNV	missense_variant	likely_pathogenic_het	1.106	Male	European(non-Finish)	0.0030476	gnomad_AF=0.0027721
92020961	SRCAP	136140	FLOATING-HARBOR SYNDROME	ad	16:30750555:G:T	SNV	missense_variant	VUS	0.866	Male	European(non-Finish)	0	no AF in gnomad
92026925	SRCAP	136140	FLOATING-HARBOR SYNDROME	ad	16:30747614:G:T	SNV	missense_variant	VUS	0.88	Female	European(non-Finish)	0	no AF in gnomad
GT04013431	STAT3	147060	HYPER-IgE SYNDROME, AUTOSOMAL DOMINANT	ad	17:40485685:G:A	SNV	splice_region_variant,intron_variant	VUS	0.694	Female	African	0	no AF in gnomad
92022422	SUFU	109400	BASAL CELL NEVUS SYNDROME	ad	10:104352413:A:G	SNV	missense_variant	VUS	0.848	Male	European(non-Finish)	0	no AF in gnomad
GT04013872	SYNE1	612998	EMERY-DREIFUSS MUSCULAR DYSTROPHY 4, AUTOSOMAL DOMINANT	ar	6:152673313:G:A	SNV	missense_variant	VUS	0.813	Male	African	0.0001148	gnomad_AF=3.1892e-05
9504000139-0	TAB2	614980	CONGENITAL HEART DEFECTS, MULTIPLE TYPES, 2	ad	6:149700430:C:A	SNV	missense_variant	VUS	1.32	Female	African	0	no AF in gnomad
9504000182-0	TAB2	614980	CONGENITAL HEART DEFECTS, MULTIPLE TYPES, 2	ad	6:149700172:C:G	SNV	missense_variant	VUS	1.505	Male	European(non-Finish)	0	no AF in gnomad
GT04014901	TAB2	614980	CONGENITAL HEART DEFECTS, MULTIPLE TYPES, 2	ad	6:149700048:C:T	SNV	missense_variant	VUS	0.812	Female	European(non-Finish)	0	no AF in gnomad
9504000026-0	TBL2	NA	NA	ad	7:72984961:G:T	SNV	missense_variant	VUS	0.858	Female	European(non-Finish)	0	NA
9504000218-0	TBX2	618223	VERTEBRAL ANOMALIES AND VARIABLE ENDOCRINE AND T-CELL DYSFUNCTION	ad	17:59485611:T:A	SNV	missense_variant	VUS	1.098	Female	Other	0	no AF in gnomad
92022371	TBX5	142900	HOLT-ORAM SYNDROME	ad	12:114793735:TG:T	INDEL	frameshift_variant	likely_pathogenic_het	3.204	Male	African	0	no AF in gnomad
92123870	TF	209300	ATRANSFERRINEMIA	ar	3:133483071:A:G	SNV	missense_variant	VUS	0.941	Male	European(non-Finish)	0	no AF in gnomad
92123870	TF	209300	ATRANSFERRINEMIA	ar	3:133496032:G:A	SNV	missense_variant	pathogenic_het	0.941	Male	European(non-Finish)	0.0041505	gnomad_AF=0.002357
9504000325-0	TGFBR1	609192	LOEYS-DIETZ AORTIC ANEURYSM SYNDROME	ad	9:101900330:G:A	SNV	missense_variant	VUS	0.746	Female	Other	0	no AF in gnomad
9504000161	TGFBR3	NA	NA	ad	1:92182263:A:G	SNV	splice_region_variant,synonymous_variant	VUS	0.805	Female	Other	0	no AF in gnomad
GT04006421	TK2	117210	SPINOCEREBELLAR ATAXIA 31	ad	16:66551041:G:A	SNV	splice_region_variant,synonymous_variant	VUS	0.915	Male	European(non-Finish)	0	no AF in gnomad
9504000196-0	TNNI3	613690	CARDIOMYOPATHY, FAMILIAL HYPERTROPHIC, 7	ad	19:55668677:C:A	SNV	splice_acceptor_variant	likely_pathogenic_het	3.541	Male	Other	0	no AF in gnomad
9504000065-0	TRIP11	184260	ODONTOCHONDRODYSPLASIA	ad	14:92470821:G:A	SNV	stop_gained	VUS	0.826	Male	European(non-Finish)	0	no AF in gnomad
GT04006322	TRRAP	618454	DEVELOPMENTAL DELAY WITH OR WITHOUT DYSMORPHIC FACIES AND AUTISM	ad	7:98592334:C:G	SNV	missense_variant	VUS	0.719	Male	European(non-Finish)	0	no AF in gnomad
92122664	TSC2	613254	TUBEROUS SCLEROSIS 2	ad	16:2126158:C:G	SNV	missense_variant	VUS	0.764	Female	European(non-Finish)	0	no AF in gnomad
GT04012532	TSC2	613254	TUBEROUS SCLEROSIS 2	ad	16:2129036:G:T	SNV	missense_variant	VUS	0.748	Male	Ashkenazi	0	no AF in gnomad
9504000084	TTR	105210	AMYLOIDOSIS, HEREDITARY, TRANSTHYRETIN-RELATED	ad	18:29178618:G:A	SNV	missense_variant	pathogenic_het	0.934	Male	African	0.017218	gnomad_AF=0.0048432
9504000161	TTR	105210	AMYLOIDOSIS, HEREDITARY, TRANSTHYRETIN-RELATED	ad	18:29172937:G:A	SNV	missense_variant	pathogenic_het	1.996	Female	Other	0	no AF in gnomad
92020505	USP8	219090	PITUITARY ADENOMA 4, ACTH-SECRETING	ad	15:50733549:T:G	SNV	stop_gained	VUS	1.384	Male	European(non-Finish)	0	no AF in gnomad
GT04013752	USP8	219090	PITUITARY ADENOMA 4, ACTH-SECRETING	ad	15:50782004:C:T	SNV	stop_gained	VUS	1.244	Female	Other	0	no AF in gnomad
92121840	WAS	301000	WISKOTT-ALDRICH SYNDROME	xr	X:48547758:C:T	SNV	missense_variant	VUS	1.228	Male	Other	0	no AF in gnomad
9504000282-0	WAS	301000	WISKOTT-ALDRICH SYNDROME	xr	X:48547758:C:T	SNV	missense_variant	VUS	1.32	Male	Other	0	no AF in gnomad
GT04014361	WT1	608978	MEACHAM SYNDROME	ad	11:32417943:C:A	SNV	missense_variant	VUS	1.18	Male	Other	0	no AF in gnomad
92021521	YWHAE	NA	NA	ad	17:1264383:T:C	SNV	splice_region_variant,intron_variant	VUS	0.821	Female	European(non-Finish)	0	no AF in gnomad
GT04008062	YWHAE	NA	NA	ad	17:1265213:C:A	SNV	missense_variant	VUS	1.433	Male	European(non-Finish)	0	no AF in gnomad

NA, not available; ad, autosomal dominant; ar, autosomal recessive; SNV, single nucleotide variant; INDEL, inserstion-deletion; het, heterozygous; VUS, variant of uncertain significance; AF, allele frequency

Supplementary Table 2. Damaging de novo genetic variants in Abnormal Heart Morphology HPO-derived genes.

PATIENT ID	GENE	LOCATION	TYPE	Consequence	AF	CLINVAR	ANCESTRY	SEX
GT04011562	CRKL	22:21288342:A:G	SNV	missense_variant	0	VUS	Other	Female
GT04010662	EXT1	8:119122874:T:A	SNV	missense_variant	0	VUS	European (non-Finish)	Male
9504000309	FANCM	14:45645935:TCA:T	INDEL	frameshift_variant	0	pathogenic_het	European (non-Finish)	Female
GT04012081	KMT2D	12:49420288:C:T	SNV	missense_variant	0	pathogenic_het	Asian	Female
GT04008173	KMT2D	12:49446763:A:T	SNV	stop_gained	0	VUS	European (non-Finish)	Female
GT04010993	KMT2D	12:49447337:G:GC	INDEL	frameshift_variant	0	VUS	European (non-Finish)	Male
GT04010722	OFD1	X:13764945:CA:C	INDEL	frameshift_variant	0	pathogenic_het	European (non-Finish)	Male
9504000208	OFD1	X:13781856:T:C	SNV	splice_region_variar	0	VUS	European (non-Finish)	Female
9504000014	OTX2	14:57271039:C:T	SNV	missense_variant	0	VUS	Ashkenazi	Male
GT04014901	RREB1	6:7248887:G:T	SNV	missense_variant	0	VUS	European (non-Finish)	Female

AF, allele frequency; VUS, variant of uncertain significance based on Clinvar classification; pathogenic_het, pathogenic heterozygous variant

Supplementary Table 3. Demographic, genetic and clinical contributions to risk of neurodevelopmental and growth outcomes.

<table>
<tr><th>Probability Query</th><th>Probability</th><th>Relative Risk</th><th>CI-5%</th><th>CI-95%</th><th>N</th><th></th><th>Probability Query</th><th>Probability</th><th>Relative Risk</th><th>CI-5%</th><th>CI-95%</th><th>N</th></tr>
<tr><td>P (MDI=Low)</td><td>0.11</td><td>-</td><td>-</td><td>-</td><td>36</td><td></td><td>P (MDI=High)</td><td>0.37</td><td>-</td><td>-</td><td>-</td><td>113</td></tr>
<tr><td>P (MDI=Low | SYNDR=Y)</td><td>0.11</td><td>1.00</td><td>1.00</td><td>1.00</td><td>7</td><td></td><td>P (MDI=High | Sex=M)</td><td>0.30</td><td>0.82</td><td>0.81</td><td>0.83</td><td>39</td></tr>
<tr><td>P (MDI=Low | dGV=Y)</td><td>0.11</td><td>1.38</td><td>1.37</td><td>1.40</td><td>20</td><td></td><td>P (MDI=High | LAZ=High)</td><td>0.46</td><td>1.22</td><td>1.21</td><td>1.23</td><td>24</td></tr>
<tr><td>P (MDI=Low | Sex=F)</td><td>0.11</td><td>1.77</td><td>1.72</td><td>1.83</td><td>26</td><td></td><td>P (MDI=High | dGV=N)</td><td>0.37</td><td>1.18</td><td>1.17</td><td>1.18</td><td>78</td></tr>
<tr><td>P (MDI=Low | LAZ=Low)</td><td>0.20</td><td>3.17</td><td>3.11</td><td>3.24</td><td>19</td><td></td><td>P (MDI=High | SYNDR=N)</td><td>0.37</td><td>2.17</td><td>2.12</td><td>2.23</td><td>115</td></tr>
<tr><td>P (MD=LowI | PDI=Low)</td><td>0.28</td><td>21.17</td><td>19.19</td><td>23.16</td><td>32</td><td></td><td>P (MDI=High | PDI=High)</td><td>0.67</td><td>2.41</td><td>2.39</td><td>2.43</td><td>37</td></tr>
<tr><td>P (MD=LowI | PDI=Low, LAZ=Low)</td><td>0.35</td><td>51.28</td><td>46.41</td><td>56.15</td><td>18</td><td></td><td>P (MDI=High | PDI=High, SYNDR=Y)</td><td>0.74</td><td>4.03</td><td>3.93</td><td>4.14</td><td>37</td></tr>
<tr><td>P (MDI=Low | PDI=Low, Sex=F)</td><td>0.40</td><td>75.79</td><td>71.04</td><td>80.54</td><td>23</td><td></td><td></td><td></td><td></td><td></td><td></td><td></td></tr>
<tr><td></td><td></td><td></td><td></td><td></td><td></td><td></td><td></td><td></td><td></td><td></td><td></td><td></td></tr>
<tr><td>Probability Query</td><td>Probability</td><td>Relative Risk</td><td>CI-5%</td><td>CI-95%2</td><td>N</td><td></td><td>Probability Query</td><td>Probability</td><td>Relative Risk</td><td>CI-5%</td><td>CI-95%</td><td>N</td></tr>
<tr><td>P (PDI=Low)</td><td>0.34</td><td>-</td><td>-</td><td>-</td><td>115</td><td></td><td>P (PDI=High)</td><td>0.16</td><td>-</td><td>-</td><td>-</td><td>50</td></tr>
<tr><td>P (PDI=Low | Sex=F)</td><td>0.24</td><td>0.61</td><td>0.60</td><td>0.61</td><td>63</td><td></td><td>P (PDI=High | Sex=M</td><td>0.19</td><td>0.97</td><td>0.95</td><td>0.99</td><td>19</td></tr>
<tr><td>P (PDI=Low | SYNDR=Y)</td><td>0.35</td><td>1.00</td><td>1.00</td><td>1.00</td><td>18</td><td></td><td>P (PDI=High | dGV=N</td><td>0.21</td><td>1.22</td><td>1.21</td><td>1.22</td><td>30</td></tr>
<tr><td>P (PDI=Low | dGV=Y)</td><td>0.35</td><td>1.16</td><td>1.15</td><td>1.17</td><td>59</td><td></td><td>P (PDI=High | LAZ=High)</td><td>0.38</td><td>2.09</td><td>2.06</td><td>2.12</td><td>15</td></tr>
<tr><td>P (PDI=Low | LAZ=Low)</td><td>0.58</td><td>2.16</td><td>2.14</td><td>2.18</td><td>58</td><td></td><td>P (PDI=High | SYNDR=N)</td><td>0.21</td><td>3.96</td><td>3.61</td><td>4.31</td><td>49</td></tr>
<tr><td>P (PDI=Low | MDI=Low)</td><td>0.91</td><td>2.90</td><td>2.88</td><td>2.92</td><td>32</td><td></td><td>P (PDI=High | MDI=High)</td><td>0.40</td><td>5.38</td><td>5.26</td><td>5.50</td><td>39</td></tr>
<tr><td>P (PDI=Low | MDI=Low, SYNDR=Y)</td><td>0.91</td><td>2.90</td><td>2.88</td><td>2.92</td><td>5</td><td></td><td>P (PDI=High | MDI=High, SYNDR=N)</td><td>0.42</td><td>8.35</td><td>7.26</td><td>9.43</td><td>39</td></tr>
<tr><td>P (PDI=Low | MDI=Low, dGV=Y)</td><td>0.92</td><td>3.12</td><td>3.10</td><td>3.15</td><td>18</td><td></td><td>P (PDI =High| MDI=High, LAZ=High)</td><td>0.48</td><td>10.77</td><td>10.40</td><td>11.15</td><td>9</td></tr>
<tr><td>P (PDI=Low | MDI=Low, LAZ=Low)</td><td>1.00</td><td>3.94</td><td>3.91</td><td>3.98</td><td>18</td><td></td><td>P (PDI=High | MDI=High, LAZ=High, SYNDR=N)</td><td>0.50</td><td>16.52</td><td>14.26</td><td>18.78</td><td>9</td></tr>
<tr><td></td><td></td><td></td><td></td><td></td><td></td><td></td><td></td><td></td><td></td><td></td><td></td><td></td></tr>
<tr><td>Probability Query</td><td>Probability</td><td>Relative Risk</td><td>CI-5%</td><td>CI-95%</td><td>N</td><td></td><td>Probability Query</td><td>Probability</td><td>Relative Risk</td><td>CI-5%</td><td>CI-95%</td><td>N</td></tr>
<tr><td>P (LAZ=Low)</td><td>0.28</td><td>-</td><td>-</td><td>-</td><td>94</td><td></td><td>P (LAZ=High)</td><td>0.18</td><td>-</td><td>-</td><td>-</td><td>56</td></tr>
<tr><td>P (LAZ=Low | dGV=Y)</td><td>0.32</td><td>1.12</td><td>1.11</td><td>1.12</td><td>45</td><td></td><td>P (LAZ=High | Sex=M)</td><td>0.15</td><td>0.94</td><td>0.94</td><td>0.95</td><td>22</td></tr>
<tr><td>P (LAZ=Low | Sex=F)</td><td>0.30</td><td>1.19</td><td>1.17</td><td>1.20</td><td>65</td><td></td><td>P (LAZ=High | dGV=N)</td><td>0.15</td><td>1.01</td><td>1.01</td><td>1.01</td><td>32</td></tr>
<tr><td>P (LAZ=Low | SYNDR=Y)</td><td>0.34</td><td>1.34</td><td>1.32</td><td>1.35</td><td>18</td><td></td><td>P (LAZ=High | SYNDR=N)</td><td>0.15</td><td>1.05</td><td>1.04</td><td>1.05</td><td>49</td></tr>
<tr><td>P (LAZ=Low | MDI=Low)</td><td>0.59</td><td>2.13</td><td>2.10</td><td>2.15</td><td>19</td><td></td><td>P (LAZ=High | MDI=High)</td><td>0.20</td><td>1.36</td><td>1.34</td><td>1.38</td><td>24</td></tr>
<tr><td>P (LAZ=Low | PDI=Low)</td><td>0.52</td><td>2.52</td><td>2.49</td><td>2.55</td><td>58</td><td></td><td>P (LAZ=High | PDI=High)</td><td>0.29</td><td>2.06</td><td>2.03</td><td>2.09</td><td>15</td></tr>
<tr><td>P (LAZ=Low | PDI=Low, dGV=Y)</td><td>0.55</td><td>2.64</td><td>2.61</td><td>2.67</td><td>31</td><td></td><td>P (LAZ=High | PDI=High, SYNDR=N)</td><td>0.28</td><td>2.05</td><td>2.02</td><td>2.09</td><td>14</td></tr>
<tr><td>P (LAZ=Low | MDI=Low, PDI=Low)</td><td>0.64</td><td>3.14</td><td>3.10</td><td>3.18</td><td>18</td><td></td><td></td><td></td><td></td><td></td><td></td><td></td></tr>
<tr><td>P (LAZ=Low | PDI=Low, SYNDR=Y)</td><td>0.76</td><td>3.83</td><td>3.79</td><td>3.88</td><td>13</td><td></td><td></td><td></td><td></td><td></td><td></td><td></td></tr>
<tr><td>P (LAZ=Low | PDI=Low, Sex=F)</td><td>0.53</td><td>6.35</td><td>5.95</td><td>6.75</td><td>31</td><td></td><td></td><td></td><td></td><td></td><td></td><td></td></tr>
<tr><td>P (LAZ=Low | PDI=Low, MDI=Low, Sex=F)</td><td>0.74</td><td>9.42</td><td>7.80</td><td>11.05</td><td>15</td><td></td><td></td><td></td><td></td><td></td><td></td><td></td></tr>
</table>

MDI, mental developmental index; PDI, psychomotor developmental index; LAZ, length for age Z-score

dGV, damaging genetic variant in genes associated with HPO term “Abnormal Heart Morphology”. SYNDR, damaging genetic variant in genes associated with syndromic CHD, defined by OMIM. CI-5%, Confidence interval 5%; CI-95%, Confidence interval, 95%

MDI, PDI Low <70; MDI, PDI High >100; LAZ low <-1.6; LAZ High >0

Supplementary Table 4. Impacts of pre- and post-operative clinical variables, genetics and demographic features on neurodevelopmental and growth outcomes.

Probability Query	Probability	Relative Risk	CI-5%	CI-95%	N
P(MDI=Low)	0.13	-	-	-	23
P(MDI=Low | Sex=F)	0.15	1.00	1.00	1.00	17
P(MDI=Low | Preterm=Y)	0.15	1.00	1.00	1.00	2
P(MDI=Low | BWT=Low)	0.15	1.00	1.00	1.00	3
P(MDI=Low | SYNDR=Y)	0.15	1.00	1.00	1.00	5
P(MDI=Low | SES=Low)	0.15	1.11	1.10	1.11	14
P(MDI=Low | LAZ=Low)	0.18	1.51	1.50	1.53	12
P(MDI=Low | dGV=Y)	0.16	1.92	1.86	1.97	14
P(MDI=Low | VENT=Y)	0.23	5.76	5.29	6.24	20
P(MDI=Low | PDI=Low)	0.35	43.54	40.89	46.20	22
P(MDI=Low | dGV=Y, VENT=Y)	0.25	10.49	9.68	11.30	12
P(MDI=Low | PDI=Low, dGV=Y)	0.36	78.62	73.20	84.04	13
P(MDI=Low | PDI=Low, VENT=Y)	0.48	273.04	242.37	303.71	19
P(MDI=Low | PDI=Low, VENT=Y, dGV=Yes)	0.49	401.15	357.88	444.42	11

Probability Query	Probability	Relative Risk	CI-5%	CI-95%	N
P(MDI=High)					
P(MDI=High | Sex=M)	0.35	1.00	1.00	1.00	21
P(MDI=High | SES=High)	0.36	1.01	1.00	1.01	30
P(MDI=High | BWT=High)	0.36	1.07	1.05	1.09	52
P(MDI=High | Preterm=N)	0.37	1.22	1.16	1.29	54
P(MDI=High | SYNDR=N)	0.37	1.74	1.49	1.99	53
P(MDI=High | VENT=N)	0.62	2.38	2.15	2.61	30
P(MDI=High | PDI=High)	0.77	2.94	2.80	3.07	19
P(MDI=High | PDI=High, VENT=N)	0.90	5.14	4.77	5.52	13
P(MDI=High | PDI=High, SYNDR=N)	0.78	5.17	4.33	6.01	19
P(MDI=High | PDI=High, SYNDR=N, Preterm=N)	0.79	14.96	9.17	20.75	18
P(MDI=High | SYNDR=N, VENT=N)	0.62	59.42	50.78	68.05	25
P(MDI=High | PDI=High, SYNDR=N, VENT=N)	0.91	140.27	119.86	160.67	13

Probability Query	Probability	Relative Risk	CI-5%	CI-95%	N
P(PDI=Low)	0.44	-	-	-	76
P(PDI=Low | Sex=F)	0.36	0.78	0.78	0.79	44
P(PDI=Low | Preterm=Y)	0.44	0.98	0.97	0.98	6
P(PDI=Low | BWT=Low)	0.43	0.99	0.99	0.99	10
P(PDI=Low | SYNDR=Y)	0.43	1.00	1.00	1.00	9
P(PDI=Low | dGV=Y)	0.45	1.11	1.11	1.12	39
P(PDI=Low | SES=Low)	0.46	1.18	1.18	1.19	44
P(PDI=Low | VENT=Y)	0.49	1.19	1.19	1.20	54
P(PDI=Low | LAZ=Low)	0.51	1.59	1.57	1.61	35
P(PDI=Low | MDI=Low)	0.99	2.68	2.66	2.70	22
P(PDI=Low | MDI=Low, SES=Low)	0.99	2.99	2.96	3.02	14
P(PDI=Low | MDI=Low, LAZ=Low)	0.99	3.41	3.37	3.45	12

Probability Query	Probability	Relative Risk	CI-5%	CI-95%	N
P(PDI=High)					
P(PDI=High | Sex=M)	0.18	1.00	1.00	1.00	8
P(PDI=High | LAZ=High)	0.20	1.16	1.13	1.19	4
P(PDI=High | BWT=High)	0.19	1.25	1.19	1.32	23
P(PDI=High | Preterm=N)	0.19	1.25	1.19	1.32	23
P(PDI=High | SYNDR=N)	0.18	1.39	1.26	1.51	23
P(PDI=High | VENT=N)	0.27	1.75	1.65	1.86	14
P(PDI=High | SES=High)	0.25	2.31	1.96	2.67	16
P(PDI=High | MDI=High)	0.39	7.49	5.84	9.14	19
P(PDI=High | SYNDR=N, SES=High)	0.33	52.76	45.64	59.87	8
P(PDI=High | MDI=High, SES=High)	0.44	164.93	146.98	182.89	11

Probability Query	Probability	Relative Risk	CI-5%	CI-95%	N
P(LAZ=Low)	0.35	-	-	-	62
P(LAZ=Low | Sex=F)	0.32	0.97	0.97	0.98	40
P(LAZ=Low | SYNDR=Y)	0.33	1.00	1.00	1.00	9
P(LAZ=Low | dGV=Y)	0.33	1.02	1.02	1.02	28
P(LAZ=Low | VENT=Y)	0.34	1.07	1.07	1.07	42
P(LAZ=Low | BWT=Y)	0.37	1.27	1.26	1.28	12
P(LAZ=Low | MDI=Low)	0.39	1.34	1.33	1.34	12
P(LAZ=Low | PDI=Low)	0.39	1.74	1.72	1.76	35
P(LAZ=Low | Preterm=Y)	0.45	1.76	1.74	1.79	8
P(LAZ=Low | SES=Low)	0.43	2.13	2.10	2.17	42
P(LAZ=Low | SES=Low, BWT=Y)	0.48	2.62	2.57	2.66	7
P(LAZ=Low | SES=Low, Preterm=Y)	0.57	3.42	3.36	3.47	4
P(LAZ=Low | SES=Low, Sex=F)	0.47	4.28	3.76	4.80	24
P(LAZ=Low | SES=Low, Sex=F, MDI=Low)	0.54	5.52	4.86	6.18	8
P(LAZ=Low | SES=Low, Sex=F, PDI=Low)	0.54	7.78	6.83	8.73	13

Probability Query	Probability	Relative Risk	CI-5%	CI-95%	N
P(LAZ=High)					
P(LAZ=High | SYNDR=N)	0.20	1.00	1.00	1.00	25
P(LAZ=High | MDI=High)	0.20	1.00	1.00	1.00	10
P(LAZ=High | VENT=N)	0.20	1.01	1.00	1.01	12
P(LAZ=High | Preterm=N)	0.20	1.09	1.07	1.11	30
P(LAZ=High | PDI=High)	0.22	1.16	1.13	1.18	4
P(LAZ=High | BWT=High)	0.20	1.18	1.14	1.22	26
P(LAZ=High | Sex=M)	0.22	1.30	1.10	1.50	14
P(LAZ=High | SES=High)	0.27	2.29	2.01	2.57	21

MDI, mental developmental index; PDI, psychomotor developmental index; LAZ, length for age Z-score

dGV, damaging genetic variant in genes associated with HPO term "Abnormal Heart Morphology". SYNDR, damaging genetic variant in genes associated with syndromic CHD, defined by OMIM. CI-5%, Confidence interval 5%; CI-95%, Confidence interval, 95%

MDI, PDI Low <70; MDI, PDI High >100; LAZ low <-1.6; LAZ High >0

Supplementary Figure 1.

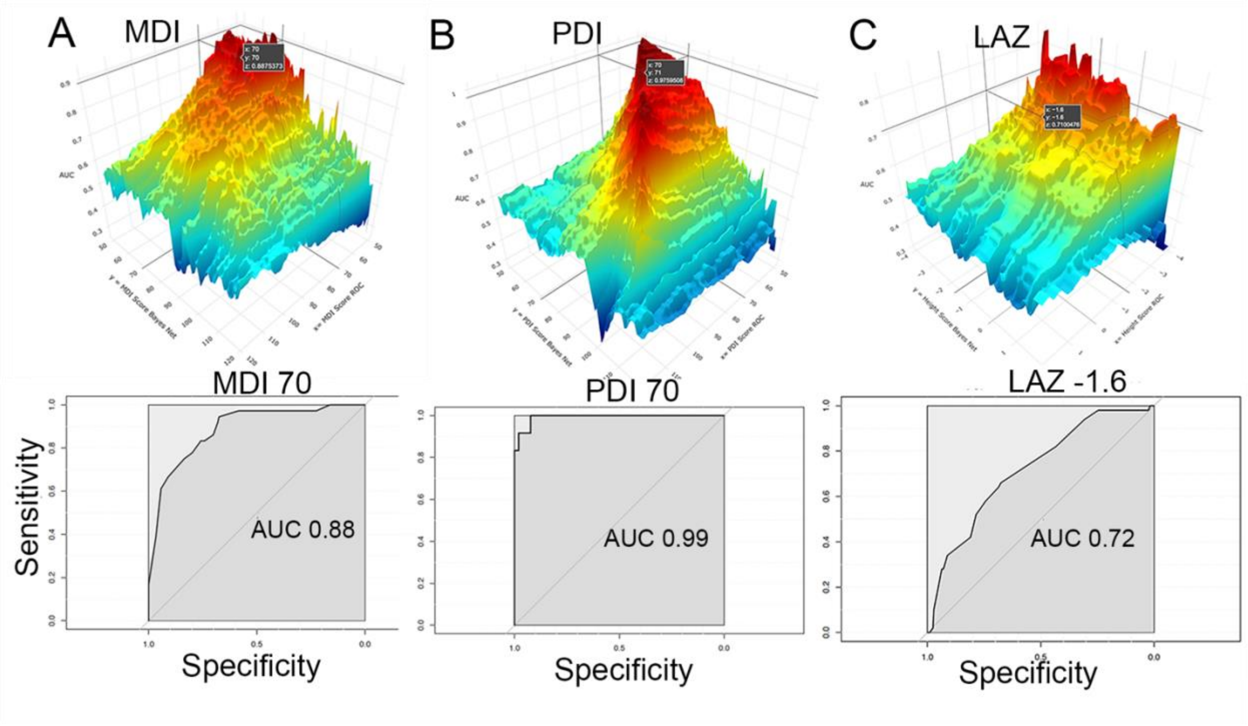

Supplementary Figure 1. Optimal discretization of continuous outcome variables. 3-dimensional heat maps for MDI (**a**), PDI (**b**) and LAZ (**c**) derived from a grid search of the nascent Bayesian Network, maximizing the area under a receiver operating curve (Z-axis), as a function of the posterior probabilities (X-axis) and continuous values (Y-axis) for each outcome variable. The receiver operator curves for the optimized values of MDI (70), PDI (70) and LAZ (-1.6 are displayed below each heat map, with their respective AUC listed. MDI, Mental Developmental Index; PDI, Psychomotor Developmental Index; LAZ, length for age z-score; AUC, area under the receiver operator curve. See text for details.

Supplementary Figure 2

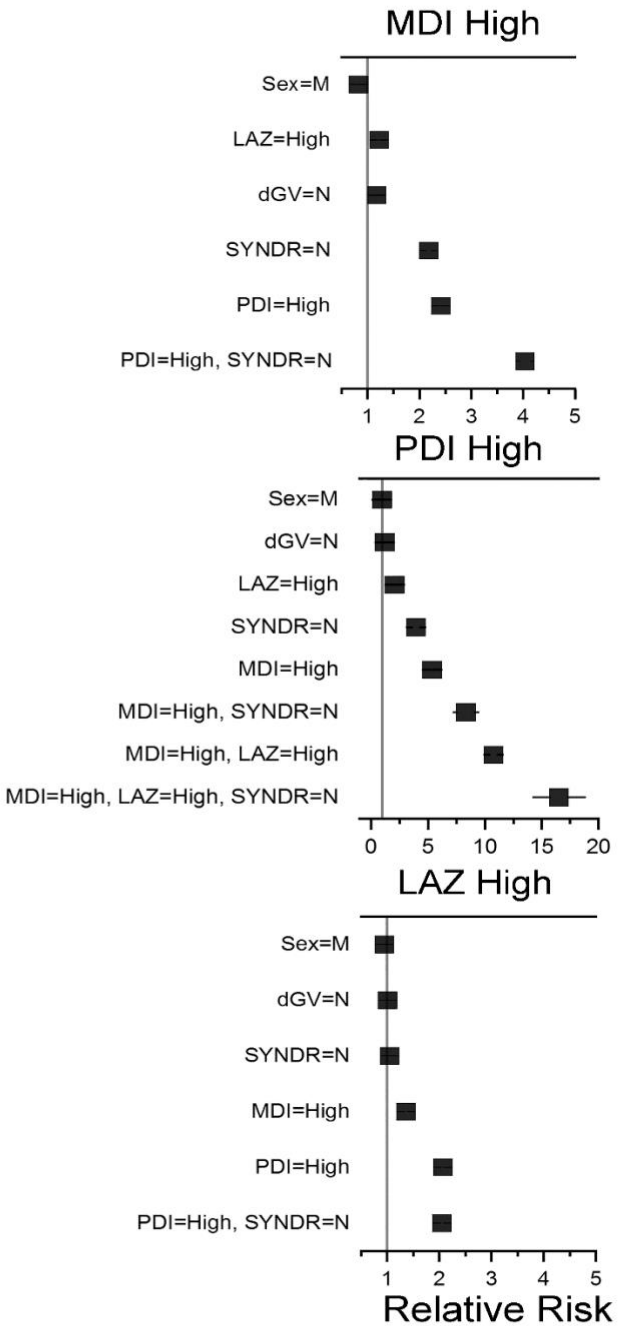

Supplementary Figure 2. Forest plots showing relative risk ratios for high MDI (>100), PDI (>100) and LAZ (>0) scores in the context of the displayed clinical variables alone or in combination. Solid black line denotes 5 and 95% confidence intervals. N=309 participants. If not visible, confidence intervals are within the symbol. SYNDR, damaging genetic variant in genes associated with syndromic CHD, defined by OMIM. MDI, mental developmental index; PDI, psychomotor developmental index; LAZ, length for age Z-score.

Supplementary Figure 3

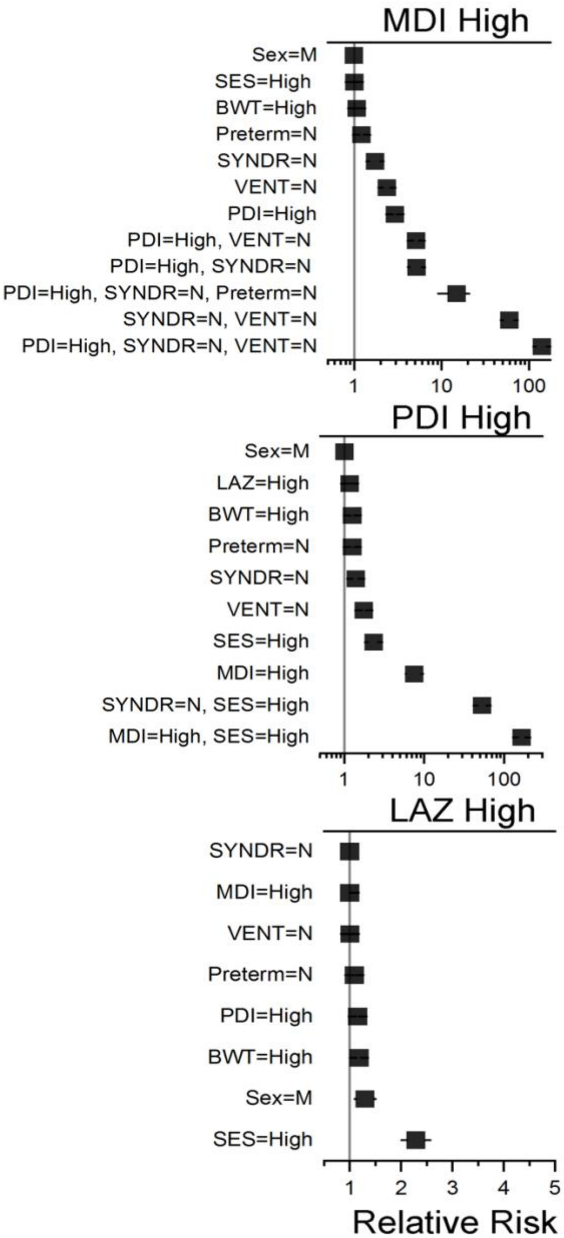

Supplementary Figure 3. Forest plots showing relative risk ratios for high MDI (>100), PDI (>100) and LAZ (>0) scores in the context of the displayed clinical variables alone or in combination. 5 and 95% confidence intervals are within the symbols. N=172 participants. If not visible, confidence intervals are within the symbol. SYNDR, damaging genetic variant in genes associated with syndromic CHD, defined by OMIM. MDI, mental developmental index; PDI, psychomotor developmental index; LAZ, length for age Z-score; BWT, birth weight; VENT, prolonged ventilation; SES, socioeconomic score; M, male.
