# Supplementary material for: Genetic and clinical variables act synergistically to impact neurodevelopmental outcomes in children with single ventricle heart disease
Source: Commun Med (Lond). 2023 Sep 27;3:127. doi: 10.1038/s43856-023-00361-2 (PMC10533527; doi:10.1038/s43856-023-00361-2)
Supplement: Supplementary file 3 — Description of Additional Supplementary Files [file 43856_2023_361_MOESM3_ESM.pdf]

Description of Additional Supplementary Files

File Name: Supplementary Data 1

Description: Raw data for primary figures
