# Supplementary material for: Genetic and clinical variables act synergistically to impact neurodevelopmental outcomes in children with single ventricle heart disease
Source: Commun Med (Lond). 2023 Sep 27;3:127. doi: 10.1038/s43856-023-00361-2 (PMC10533527; doi:10.1038/s43856-023-00361-2)
Supplement: Supplementary file 5 — NR reporting summary [file 43856_2023_361_MOESM5_ESM.pdf]

nature portfolio

Corresponding author(s): Thomas A Miller

Last updated by author(s): Feb 28, 2023

Reporting Summary

Nature Portfolio wishes to improve the reproducibility of the work that we publish. This form provides structure for consistency and transparency in reporting. For further information on Nature Portfolio policies, see our Editorial Policies and the Editorial Policy Checklist.

Statistics

For all statistical analyses, confirm that the following items are present in the figure legend, table legend, main text, or Methods section.

n/a	Confirmed	
☐	☒	The exact sample size (n) for each experimental group/condition, given as a discrete number and unit of measurement
☐	☒	A statement on whether measurements were taken from distinct samples or whether the same sample was measured repeatedly
☐	☒	The statistical test(s) used AND whether they are one- or two-sided<br>*Only common tests should be described solely by name; describe more complex techniques in the Methods section.*
☐	☒	A description of all covariates tested
☐	☒	A description of any assumptions or corrections, such as tests of normality and adjustment for multiple comparisons
☐	☒	A full description of the statistical parameters including central tendency (e.g. means) or other basic estimates (e.g. regression coefficient) AND variation (e.g. standard deviation) or associated estimates of uncertainty (e.g. confidence intervals)
☒	☐	For null hypothesis testing, the test statistic (e.g. F, t, r) with confidence intervals, effect sizes, degrees of freedom and P value noted<br>*Give P values as exact values whenever suitable.*
☐	☒	For Bayesian analysis, information on the choice of priors and Markov chain Monte Carlo settings
☐	☒	For hierarchical and complex designs, identification of the appropriate level for tests and full reporting of outcomes
☐	☒	Estimates of effect sizes (e.g. Cohen's d, Pearson's r), indicating how they were calculated

Our web collection on statistics for biologists contains articles on many of the points above.

Software and code

Policy information about availability of computer code

Data collection	PHN and PCGC data availability can be found at https://www.pediatricheartnetwork.org/login/ and https://benchtobassinet.com/?page_id=141
Data analysis	Analytic approaches and programing are available in previously published articles that are referenced throughout the manuscript with links provided where appropriate

For manuscripts utilizing custom algorithms or software that are central to the research but not yet described in published literature, software must be made available to editors and reviewers. We strongly encourage code deposition in a community repository (e.g. GitHub). See the Nature Portfolio guidelines for submitting code & software for further information.

Data

Policy information about availability of data

All manuscripts must include a data availability statement. This statement should provide the following information, where applicable:

- Accession codes, unique identifiers, or web links for publicly available datasets
- A description of any restrictions on data availability
- For clinical datasets or third party data, please ensure that the statement adheres to our policy

The sequencing data used in this analysis may be downloaded, with committee-approved access, from the database of Genotypes and Phenotypes (dbGaP) [www.ncbi.nlm.nih.

gov/] (accession numbers phs000571.v5.p2). Clinical data for this cohort is available as a public use dataset (https://www.pediatricheartnetwork.org/login/).

Human research participants

Policy information about studies involving human research participants and Sex and Gender in Research.

Reporting on sex and gender	Results based on differences in sex are reported in this study. Gender data are not available for these cohorts.
Population characteristics	The population in this study are two previously described clinical trial cohorts.
Recruitment	This is a secondary analysis of two previously recruited cohorts with new linking between phenotype and genotype data, not available in the initial study. There is survival bias in this analytic cohort as DNA collection was not a requirement in the initial enrollment, and some subjects had DNA collected later.
Ethics oversight	IRBs from all centers participating in the Pediatric Heart Network trials. Secondary analysis was approved by the PHN and Pediatric Cardiac Genomics Consortium

Note that full information on the approval of the study protocol must also be provided in the manuscript.

Field-specific reporting

Please select the one below that is the best fit for your research. If you are not sure, read the appropriate sections before making your selection.

☒ Life sciences ☐ Behavioural & social sciences ☐ Ecological, evolutionary & environmental sciences

For a reference copy of the document with all sections, see nature.com/documents/nr-reporting-summary-flat.pdf

Life sciences study design

All studies must disclose on these points even when the disclosure is negative.

Sample size	Determined by those enrolled in SVR and ISV trials who had length measurement and developmental testing at 14 months as well as whole exome sequencing.
Data exclusions	No data were excluded. Exclusion criteria for the initial trials have been previously described.
Replication	A validation cohort was not included in this preliminary work, proof of principle work. Unfortunately there are very few CHD cohorts that are so well phenotyped and genotyped. PCGC and PHN investigators are currently working on creating additional cohorts for such validation.
Randomization	Randomization is not relevant to this study as the randomization in the original trials were not associated with the outcomes of interest described in this study
Blinding	Investigators were blinded to the randomization arms in the original trials.

Reporting for specific materials, systems and methods

We require information from authors about some types of materials, experimental systems and methods used in many studies. Here, indicate whether each material, system or method listed is relevant to your study. If you are not sure if a list item applies to your research, read the appropriate section before selecting a response.

Materials & experimental systems

n/a	Involved in the study	
☒	☐	Antibodies
☒	☐	Eukaryotic cell lines
☒	☐	Palaeontology and archaeology
☒	☐	Animals and other organisms
☐	☒	Clinical data
☒	☐	Dual use research of concern

Methods

n/a	Involved in the study	
☒	☐	ChIP-seq
☒	☐	Flow cytometry
☒	☐	MRI-based neuroimaging

Clinical data

Policy information about clinical studies

All manuscripts should comply with the ICMJE guidelines for publication of clinical research and a completed CONSORT checklist must be included with all submissions.

Clinical trial registration	SVR (NCT00115934) and the ISV (NCT 00113087)
Study protocol	Ohye, R.G. et al. Design and rationale of a randomized trial comparing the Blalock-Taussig and right ventricle-pulmonary artery shunts in the Norwood procedure. J Thorac Cardiovasc Surg 136, 968-75 (2008).<br><br>Hsu, D.T. et al. Rationale and design of a trial of angiotensin-converting enzyme inhibition in infants with single ventricle. Am Heart J 157, 37-45 (2009).
Data collection	Ohye, R.G. et al. Design and rationale of a randomized trial comparing the Blalock-Taussig and right ventricle-pulmonary artery shunts in the Norwood procedure. J Thorac Cardiovasc Surg 136, 968-75 (2008).<br><br>Hsu, D.T. et al. Rationale and design of a trial of angiotensin-converting enzyme inhibition in infants with single ventricle. Am Heart J 157, 37-45 (2009).
Outcomes	Ohye, R.G. et al. Design and rationale of a randomized trial comparing the Blalock-Taussig and right ventricle-pulmonary artery shunts in the Norwood procedure. J Thorac Cardiovasc Surg 136, 968-75 (2008).<br><br>Hsu, D.T. et al. Rationale and design of a trial of angiotensin-converting enzyme inhibition in infants with single ventricle. Am Heart J 157, 37-45 (2009).
